# Development and field evaluation of a low-cost fuzzy logic irrigation controller for vineyards in semi-arid regions

**DOI:** 10.1038/s41598-026-56154-9

**Published:** 2026-06-03

**Authors:** Shayesteh Tabatabaei

**Affiliations:** Faculty of Multimedia, Tabriz Islamic Art University, Tabriz, Iran

**Keywords:** Smart irrigation, Fuzzy logic, Vineyard, Water use efficiency, Arduino, Sensor-based control, Engineering, Environmental sciences, Hydrology

## Abstract

Water scarcity in arid and semi-arid regions calls for irrigation strategies that adapt to real-time field conditions. This pilot study presents a low-cost fuzzy logic–based irrigation controller for vineyards in Malekan County (East Azerbaijan, Iran). The controller uses three environmental inputs (soil moisture, air temperature, and a solar-radiation index derived from light intensity (LDR)) and applies a rule-based fuzzy inference system to determine irrigation duration (with pump activation derived from the duration). A rainfall-gating rule is also used to prevent unnecessary irrigation during rainfall events. The system was implemented on an Arduino Uno using off-the-shelf sensors and evaluated through MATLAB surface analysis and a field pilot conducted from March to August 2024. In addition, an adjacent-plot pilot comparison against conventional irrigation was performed to examine seasonal water use and plot-level grape performance indicators (cluster weight, °Brix, acidity, and rotten cluster rate). The proposed controller was associated with a 34.6% reduction in irrigation water use (520 → 340 m^3^/ha) and descriptive improvements in cluster weight, °Brix, and rotten cluster incidence. These pilot results demonstrate the technical feasibility and operational reliability of the fuzzy logic–based controller under real-world semi-arid vineyard conditions, alongside promising descriptive improvements in water-use efficiency and key grape performance indicators. However, as this was a single-season, unreplicated adjacent-plot pilot study, the observed differences in yield and quality parameters should be interpreted as exploratory. Larger-scale, multi-season field trials are therefore required to statistically validate these preliminary findings and confirm their generalizability.

## Introduction

Water, as one of the essential resources in agriculture, plays a fundamental role in ensuring food security and environmental sustainability. Water consumption is a critical issue in all regions, particularly in arid and desert farmlands^1^. Without proper water resource management, water scarcity will become a significant obstacle to national development^2^. Therefore, effective irrigation management and scheduling are essential to reduce water consumption and improve water use efficiency in agricultural lands^3^. A marked portion of water waste is due to the use of traditional irrigation methods^4^. In arid and semi-arid regions such as East Azerbaijan Province, optimal water resource management-especially in the agricultural sector-is of great importance. Malekan County, as one of the major grape-producing regions in Iran, faces serious challenges in meeting the irrigation demands of its vineyards. The increasing demand for agricultural production, coupled with declining water resources, has highlighted the urgent need to adopt modern technologies. Traditional irrigation systems, due to their low efficiency and considerable water losses, no longer meet the needs of modern agriculture. Hence, developing intelligent irrigation systems that can manage water application accurately and efficiently based on environmental conditions and the actual water needs of plants is essential. In recent years, considerable research has been conducted in this regard, and some of these studies will be discussed below. Alves et al.^5^ introduced a digital twin framework for smart irrigation systems aimed at enhancing agricultural productivity and reducing water consumption. This system enables optimized irrigation management by establishing an automated bidirectional data flow between physical components and their virtual counterparts. By integrating physical sensors and actuators with an IoT platform based on FIWARE, it collects and processes data related to soil conditions, weather, and crop status. This approach allows farmers to simulate and evaluate various irrigation strategies before implementation, thus improving decision-making, reducing water usage, and optimizing agricultural operations. However, it also faces challenges such as the need for advanced technical expertise, high initial costs, and limitations in scalability and adaptability across diverse agricultural environments. Krishnan et al.^6^ proposed a smart irrigation system based on fuzzy logic and the Internet of Things (IoT) to optimize water usage and improve agricultural efficiency. This system monitors key environmental parameters, including soil moisture, temperature, and humidity, through integrated sensors and IoT controllers. Fuzzy logic is employed to process the data and determine precise irrigation requirements, ensuring that water is applied only when necessary. Experimental evaluations indicated marked water savings and improved crop health. Nevertheless, the system faces limitations such as dependency on stable internet connectivity, high initial implementation costs, and scalability challenges in larger or more heterogeneous agricultural settings. Ben Yezza et al.^7^ introduced an IoT-based smart irrigation system utilizing fuzzy control technology to optimize water and energy usage in agriculture. The system uses a wireless sensor network (WSN) to gather data such as soil moisture and temperature across different zones of a greenhouse and transmits it via radio communication to a central server. A fuzzy logic controller processes the data to make intelligent irrigation decisions. With a user interface developed using Node-RED, remote monitoring and control of irrigation are possible at any time and from any location. The system was implemented in a real-world scenario for tomato cultivation, showing a substantial reduction in both water and energy consumption. Morchid et al.^8^ presented an IoT-based smart irrigation management system designed to enhance agricultural water security through the integration of embedded systems, telemetry data, and cloud computing. The system architecture comprises three layers: IoT devices, the ThingsBoard cloud platform, and a user dashboard that supports data flow, secure communication, and user interaction. Environmental data such as temperature, humidity, soil moisture, and water level are collected by sensors and processed using a fuzzy logic controller to make smart irrigation decisions. Results suggest the system’s effectiveness in improving agricultural sustainability, optimizing water usage, and supporting intelligent farming practices. By combining embedded systems, IoT, cloud computing, and advanced algorithms, this approach contributes to maximizing agricultural productivity and ensuring global food and water security. Et-taibi et al.^9^ developed a smart irrigation system leveraging cloud computing and IoT technologies to enhance water management in smart agriculture. The system employs embedded systems, telemetry, and cloud-based processing to optimize irrigation processes. It features a three-layer architecture comprising IoT devices, the ThingsBoard cloud platform, and a user dashboard that facilitates secure data transmission and interaction. Environmental variables such as temperature, humidity, soil moisture, and water level are collected by sensors and processed by a fuzzy logic controller to make optimal irrigation decisions. Additionally, the system enables remote monitoring and control via a Node-RED-based user interface. Implementation in a real-world setting revealed marked reductions in water and energy consumption compared to traditional irrigation methods. Lephondo et al.^10^ proposed a smart irrigation system based on machine learning to optimize water usage in agriculture. This system employs IoT technology to gather real-time data on soil moisture, weather conditions, and crop water requirements. By applying machine learning algorithms, the system analyzes the data to forecast optimal irrigation schedules. The study demonstrated the system’s effectiveness in significantly reducing water consumption while providing farmers with real-time information on soil moisture, weather APIs, varying crop water needs, and sensor-driven irrigation predictions. Singh et al.^11^ conducted a three-year field study (2019–2021) in Irvine, California, evaluating the performance of a smart soil-moisture-sensor-based controller for automating the irrigation of hybrid Bermuda grass using recycled water. The study tested 12 irrigation strategies combining six soil moisture thresholds and two irrigation methods (regulated vs. demand-based). The impact of these strategies on turf quality was assessed using weekly NDVI data and canopy temperature during the summer irrigation season. Soil salinity and sodium adsorption ratio (SAR) were also measured before and after the irrigation season. Findings showed that irrigation levels and frequency constraints significantly affected NDVI over the three years. Canopy-air temperature differences were influenced by irrigation levels, with frequency constraints showing marked effects in 2021 and 2022. Demand-based irrigation reduced temperature differences by 10% compared to regulated irrigation. Although soil salinity fluctuated during testing periods, both salinity and SAR increased over time. The study concluded that full automation of hybrid Bermuda grass irrigation using recycled water at 75% field capacity thresholds in a semi-arid climate may result in suboptimal turf quality. Liopa-Tsakalidi et al.^12^ introduced a LoRaWAN-based smart irrigation platform tailored for olive orchards. The system utilizes low-cost microcontrollers and embedded sensors to monitor soil moisture at various depths. Collected data is transmitted to a cloud infrastructure via LoRaWAN. Deployed in a 22-hectare olive orchard in Greece, the platform demonstrated a 42% and 25% reduction in water usage in 2020 and 2021, respectively. The research highlights the potential of advanced technologies such as LoRaWAN in improving agricultural water management and enhancing resource efficiency. Ala’F et al.^13^ introduced an IoT-based environmental sensing and prediction model for intelligent irrigation systems. The model utilizes an enhanced wind-driven optimization (EWDO) algorithm integrated with a least squares support vector machine (LSSVM) to optimize irrigation timing and water volume based on environmental sensor data and climate forecasts. The results showed that this approach effectively improves irrigation scheduling and water usage efficiency, thereby supporting sustainable agricultural productivity. The study emphasizes that advanced optimization algorithms combined with environmental sensing can substantially enhance the performance of smart irrigation systems and promote efficient water resource management in agriculture. Zhang and Li^14^ presented an IoT-based intelligent and automated irrigation system integrated with authentication mechanisms over vehicular ad hoc networks (VANETs). The system incorporates an energy-aware fuzzy routing algorithm and neural networks to optimize irrigation strategies using real-time sensor data, such as soil moisture and temperature. Key agreement mechanisms within the VANET framework ensure secure and authenticated communication between devices, safeguarding the integrity and confidentiality of irrigation data and control commands. This integration allows users to monitor and manage the irrigation system remotely through mobile devices such as smartphones and computers, enabling real-time information access and control. Although previous researches show the potential of intelligent irrigation control strategies using sensor systems, such techniques are subject to constraints such as high cost of implementation, requiring permanent access to the internet and cloud infrastructure, and inadequate verification under practical conditions in orchards. To bridge this scientific gap, in the present study, an intelligent orchard irrigation controller using fuzzy logic with low operating costs for orchards in Malekan county in the east Azerbaijan province has been developed. Fuzzy logic controllers are particularly well-suited for vineyard irrigation in resource-constrained environments due to their inherent robustness to sensor noise and measurement uncertainty, extremely low computational and memory requirements (fully compatible with low-cost microcontrollers such as the Arduino Uno), physiologically interpretable if–then rule bases that do not rely on weather forecasts or reference evapotranspiration data, and a transparent decision-making process that significantly increases farmer trust and adoption in rural areas. By effectively handling the inherent nonlinearity and uncertainty of key agricultural variables (soil moisture, temperature, and solar radiation) using simple, expert-derived rules, the proposed approach delivers more reliable performance where complex alternatives would be impractical or prohibitively expensive. The proposed system consists of three inputs of soil moisture, air temperature, and sunshine index on the platform of Arduino Uno through sensors by implementing 27 fuzzy rules that include one raindrop regulation rule that avoids unnecessary irrigation during rainfall and after that. The proposed controller was tested in a real orchard, and then, a comparison was made in the adjacent plot with traditional irrigation in terms of water consumption and quality parameters of grapes.

Although fuzzy logic-based irrigation controllers have been reported in the literature, the present pilot study is distinguished by an unprecedented combination of ultra-low cost (< US$45 total), complete offline operation, first-ever deployment in a traditional Iranian bush-vine raisin grape vineyard, integration of a calibrated LDR-based solar-radiation proxy, and full-season field validation that achieved 34.6% water savings with descriptive improvements in grape quality. This unique integration delivers a genuinely practical, replicable, and farmer-accessible solution for smallholder vineyards in developing semi-arid regions — a critical real-world context that remains largely unaddressed in previous works.

The main contributions of this pilot study are fourfold. First, it presents the design and implementation of an ultra-low-cost, fully standalone Arduino-based fuzzy irrigation controller specifically tailored to semi-arid vineyard conditions. Second, it provides a physiologically justified 27-rule fuzzy inference model with clearly defined membership functions that effectively integrates soil moisture, air temperature, and an LDR-derived solar-radiation index to determine irrigation duration. Third, it reports the first documented full-season field deployment and validation of such a system in a traditional bush-vine raisin grape vineyard. Fourth, it demonstrates a 34.6% reduction in seasonal irrigation water use accompanied by descriptive improvements in key grape quality parameters (cluster weight, °Brix, acidity, and rotten cluster percentage) compared with conventional farmer practice in an adjacent plot.

The remainder of the paper is organized as follows:

 “Introduction” section presents the related work. “Materials and methods” section introduces the proposed method. “Simulation of the proposed method” section describes the simulation of the proposed system using MATLAB. Finally, “Conclusion” section concludes the paper.

## Materials and methods

This section first outlines the research assumptions, followed by the objectives, and finally describes the hardware components of the proposed system.

### Study site and period

**Fig. 1:**
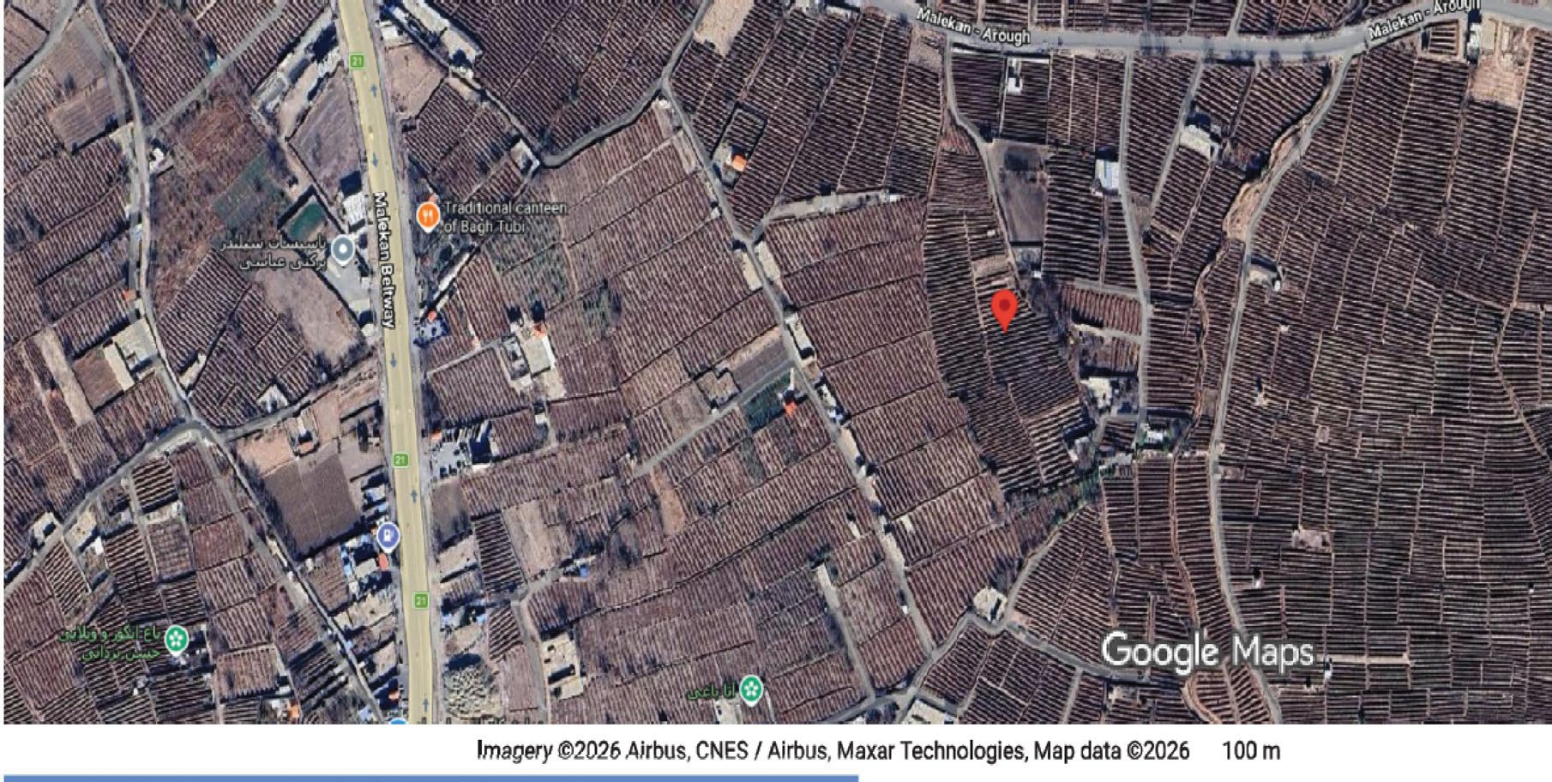
Geographic location of the study site in Malekan County, East Azerbaijan Province, Iran (37°08′01.5″N, 46°07′19.1″E), showing the experimental 1-ha vineyard plot (red marker). Map data courtesy of Google (2026); Imagery courtesy of Airbus, CNES/Airbus, and Maxar Technologies (2026). Retrieved from Google Maps, April 2026. Available at: https://www.google.com/maps/place/37°08’01.5"N+46°07’19.1"E.

The field study was conducted in a commercial vineyard located in Malekan County, East Azerbaijan Province, Iran, at coordinates 37.13375°N and 46.12197°E, with an average elevation of approximately 1302 m above sea level. The study area has a cold semi-arid climate (BSk according to the Köppen climate classification), with an annual precipitation ranging from 280 to 320 mm, the majority of which falls outside the grapevine growing season. During the study period, daily air temperatures varied from 12 °C in early spring to 38 °C in mid-summer. The dominant cultivar in both the treatment and control plots was ‘Bidaneh Keshmeshi’ (syn. Keshmeshi Sefid), a widely grown raisin grape in the region that exhibits considerable sensitivity to water stress and fungal diseases associated with excessive or poorly timed irrigation. The vines were planted at a spacing of 2.5 m × 2.5 m (approximately 1600 vines ha⁻^1^). In this traditional vineyard system, the vines are trained as individual, free-standing bush vines without any trellis or wire support. The canes are laid prostrate on small mounds, a practice widely adopted for raisin grape cultivars in the Malekan region due to its low cost, simplicity, and excellent adaptation to local climatic conditions. Figure 1 presents a satellite image of the study vineyard, with the red pin indicating the center of the experimental 1-ha plot (Google Earth, accessed April 2026).

### Sensor calibration and measured data

All sensors were calibrated in situ prior to deployment using vineyard-specific soil parameters and local environmental conditions.

Soil moisture sensor: A capacitive soil moisture sensor was employed. Two-point calibration was performed using soil samples collected from the root zone (0–50 cm depth):

Dry point (0%): oven-dried soil (110 °C for 24 h) → ADC reading ${A}_{dry}$ = 1023.

Wet point (100%): saturated soil at field capacity → ADC reading ${A}_{Wet}$ = 420.

Soil moisture percentage was calculated as Eq. 1:

1 $$\mathrm{S}\mathrm{o}\mathrm{i}\mathrm{l} \mathrm{M}\mathrm{o}\mathrm{i}\mathrm{s}\mathrm{t}\mathrm{u}\mathrm{r}\mathrm{e} \left(\mathrm{\%}\right)=100\times \frac{{\mathrm{A}}_{\mathrm{d}\mathrm{r}\mathrm{y}}-\mathrm{A}}{{\mathrm{A}}_{\mathrm{d}\mathrm{r}\mathrm{y}}-{\mathrm{A}}_{\mathrm{w}\mathrm{e}\mathrm{t}}}$$

where A is the real time ADC reading (0–1023).

solar-radiation index derived from light intensity (LDR): A low-cost solar-radiation index derived from light intensity (LDR) connected in a voltage divider circuit was used as an inexpensive proxy for solar radiation intensity. The sensor was calibrated in situ on a clear-sky day (10 March 2024) under full sunlight conditions against a reference pyranometer (Apogee SP-110, accuracy ± 5%). Both the LDR and the pyranometer were mounted horizontally at the approximate canopy height of the bush vines (1.65 m above ground level) and oriented identically. Paired measurements were recorded simultaneously at 5-min intervals from 06:30 to 17:00 local time, resulting in a total of 126 valid data pairs. A strong linear relationship was obtained (R^2^ = 0.96). Based on the calibration equation, the full ADC range (0–1023) was divided into three qualitative solar-radiation classes directly used in the fuzzy inference system: < 300 (low), 300–600 (medium), and > 600 (high radiation).

Air Temperature Sensor: Air temperature was measured using a DS18B20 digital temperature sensor with a nominal accuracy of ± 0.5 °C. The sensor was calibrated in a controlled water bath against a certified mercury thermometer at five temperature points ranging from 5 to 45 °C. The maximum observed deviation during calibration was 0.3 °C. It was installed inside a passive radiation shield at a standard height of 1.65 m above ground level to minimize the effects of direct solar radiation and soil heat.

Rain sensor and rainfall-gating rule: A resistive rain detection module (FC-37) was employed and configured to output a digital HIGH signal upon detection of rain droplets, corresponding to an approximate rainfall intensity exceeding 0.2 mm within a 15-min period. Additionally, a dedicated rainfall-gating rule was implemented in the controller firmware such that, whenever the rain sensor was triggered or cumulative daily rainfall exceeded 0 mm, pump activation was unconditionally disabled for the subsequent 24 h, irrespective of the fuzzy logic output. This rule functioned as an override to the fuzzy inference system. Given the pilot-scale nature of the study and the limited internal data-logging capacity of the Arduino Uno, continuous high-frequency raw sensor data storage was not feasible. Instead, instantaneous sensor readings were manually recorded at approximately 12:00 local time on selected representative dates throughout the growing season (March–August 2024). These spot measurements, together with the corresponding controller outputs (pump status and irrigation duration), are reported in Table 2 to illustrate system performance across a wide range of environmental conditions.

Pump flow rate verification: The irrigation pump (4.8 m^3^ h⁻^1^ nominal) was verified in situ using the volumetric method (three 10-min trials), yielding an actual flow rate of 4.81 ± 0.12 m^3^ h⁻^1^.

All calibration parameters remained stable throughout the deployment period, with spot checks in May and July 2024 showing drift < 2% for soil moisture and < 3% for the LDR-based index.

## Research assumptions

This study was based on three key assumptions: (i) a smart irrigation system can substantially improve water-use efficiency in vineyards; (ii) irrigation duration can be effectively optimized using real-time measurements of soil moisture, ambient temperature, solar-radiation index, and rainfall; and (iii) adjusting irrigation timing according to solar radiation intensity reduces evaporative losses.

## Research objectives

The primary objective of this research is to design a smart irrigation system using fuzzy logic to manage water delivery to vineyards. This system aims to determine irrigation timing and frequency while controlling the amount of water used. A key feature is its ability to prevent water evaporation by accurately adjusting the irrigation schedule.

### Scope and significance

Efficient irrigation management plays a crucial role in the growth, yield, and quality of grapes. Various irrigation techniques have been developed to optimize water usage while maintaining plant health, considering factors such as soil moisture, climate conditions, and the physiological needs of the plant. Common vineyard irrigation methods include drip irrigation, sprinkler irrigation, surface irrigation, and smart irrigation systems based on sensor networks. Among these, drip irrigation is considered one of the most effective and widely used methods due to its ability to reduce evaporation and runoff, enhance water efficiency, and maintain consistent soil moisture. In this method, water is delivered directly and precisely to the root zone, which not only improves plant growth but also reduces weed proliferation and the risk of fungal diseases. Sprinkler systems are typically used in areas with sufficient water resources. These systems can be either mobile or fixed, providing uniform water coverage across the vineyard. However, their efficiency may decline under windy conditions or high temperatures. Nevertheless, they are effective for cooling vineyards in hot climates. In surface irrigation, water flows through channels or basins between the vines and infiltrates the soil to meet the plant’s moisture needs. This method is more suitable for heavy soils and gently sloped lands but generally offers lower water use efficiency than other methods. To improve its performance, flow control systems and soil moisture monitoring technologies can be integrated. The choice of an appropriate irrigation method depends on climate conditions, soil type, and access to advanced technologies. Modern methods such as drip irrigation and sensor-based smart systems can significantly reduce water consumption and boost grape production efficiency. In many regions, vineyards are located at considerable distances from farmers’ residences. As a result, farmers must make several trips daily to manually control irrigation, leading to increased operational costs, time loss, and unnecessary water consumption. To address this challenge, this study proposes a smart irrigation system for efficient water management in the vineyards of East Azerbaijan Province. The system operates autonomously and is characterized by low cost, high irrigation accuracy, water-saving capabilities, and easy accessibility of its components.To evaluate the system’s precision, an Arduino-based microcontroller platform is employed, which receives environmental data through connected sensors. Based on the collected data, the system automatically decides whether to activate or deactivate the water pump.

### System components

**Fig. 2:**
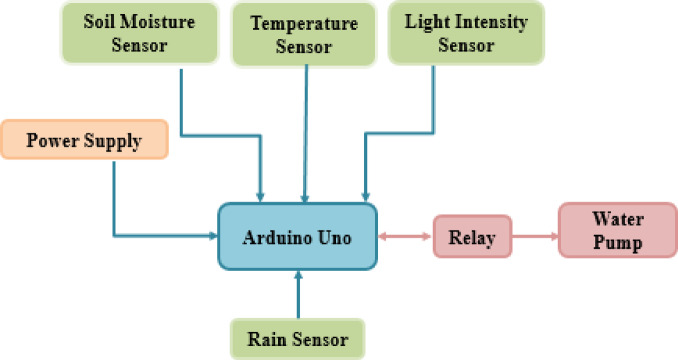
Main Components of the Proposed Smart Irrigation System.

The proposed irrigation system consists of two main components, as illustrated in Fig. 2:

- **Coordinator node**: This includes the Arduino microcontroller, which serves as the central processing unit.
- **End device node**: This comprises the following elements:
  - A water pump to supply the required water to the vineyard.
  - A soil moisture sensor to monitor the moisture level in the soil.
  - A temperature sensor to measure the ambient temperature.
  - A rain sensor to detect rainfall conditions.
  - A solar-radiation index derived from light intensity (LDR) sensor to measure the intensity of solar radiation.
- **Sensor specifications and sampling**

 Each sensor was interfaced with the Arduino Uno using analog/digital inputs. Sensor readings were acquired at a fixed sampling interval (every 15 min) and used for real-time irrigation decisions. Due to the pilot nature of the deployment and limited data retention, only representative field observations are reported in Table 2.

**Fig. 3:**
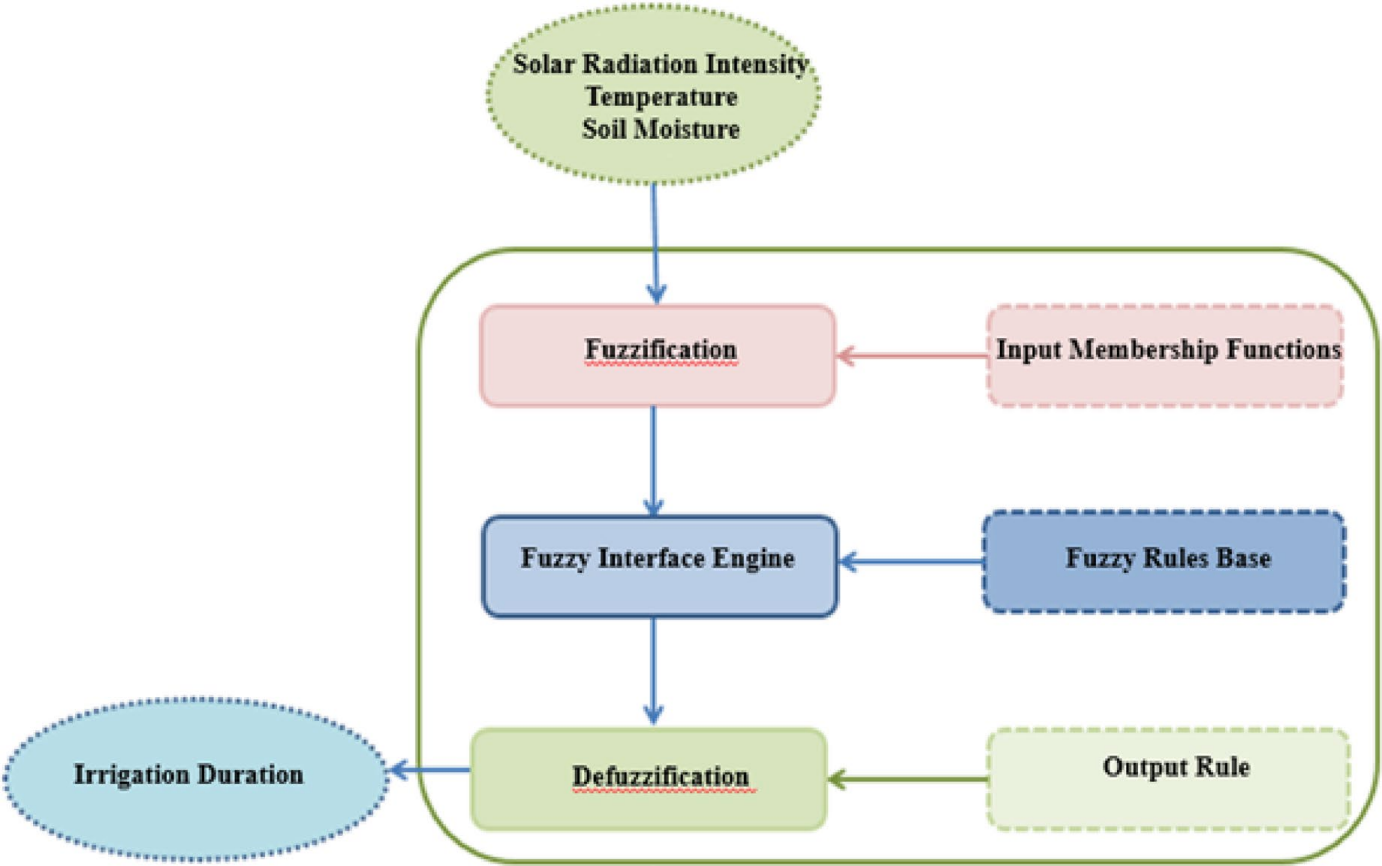
Structure of the fuzzy inference system.

In this system, the Arduino microcontroller receives and processes environmental data collected through the sensors. Based on real-time vineyard conditions, it adjusts the operation of the water pump and determines the irrigation duration accordingly. The overall structure of the fuzzy inference system is shown in (Fig. 3).

### System operation mechanism

The system operates by collecting environmental data and making irrigation decisions using fuzzy logic. The overall operational process is described as follows:

- **System activation**: Once the power supply is turned on, the Arduino microcontroller begins processing sensor data.
- **Data acquisition and processing**:
  - **solar-radiation index derived from light intensity (LDR)**: The LDR provides an analog reading (0–1023). Depending on the sensor circuit configuration, the raw reading may increase or decrease with light intensity. In this study, the LDR signal was used as a solar-radiation index for the fuzzy controller, where higher index values represent higher solar-radiation conditions after calibration/mapping.
  - **Soil moisture sensor**: The soil moisture sensor provides an analog output (0–1023). To report soil moisture in percentage, a two-point calibration was performed using (i) air-dry soil and (ii) fully saturated soil collected from the vineyard. Let A_dry_ and A_wet_ denote the corresponding ADC readings. Soil moisture percentage was computed as Eq. 1:
    2 $$SM(\% ) = 100 \times \frac{{A_{dry} - A}}{{A_{dry} - A_{wet} }}$$
    where A is the real-time ADC reading. After calibration, soil moisture was expressed in percentage and used directly as an input to the fuzzy inference system. Irrigation duration was determined by the fuzzy rules, while rainfall gating could override irrigation.
  - **Temperature Sensor**: This is used to determine the influence of ambient temperature on the vineyard’s irrigation needs.
  - **Rain sensor**: When rainfall is detected, the system disables the water pump to prevent unnecessary irrigation.
  - **Rainfall gating rule:** If rainfall was detected (rain sensor ON or rainfall > 0 mm), irrigation was disabled for a predefined lockout period (24 h) to avoid unnecessary watering. This gating condition overrides fuzzy outputs.
- **Fuzzy logic decision-making**: Based on the input values and predefined fuzzy rules, the microcontroller evaluates the irrigation requirements and issues commands to activate or deactivate the pump accordingly.

### Application of fuzzy logic in decision-making

The proposed system utilizes fuzzy logic to analyze input data and make optimized irrigation decisions. This approach is particularly effective in environments characterized by data uncertainty and variability.

#### Fuzzy variables

The fuzzy inference system includes three input variables and one output variable:

- **Inputs**
  - Soil Moisture (Dry, Moderate, Wet).
  - Ambient Temperature (Cold, Warm, Hot).
  - Light Intensity (Low, Medium, High).
- **Output** Irrigation duration (minutes). Pump status is derived as ON if duration > 0, otherwise OFF.

#### Fuzzy rules

A comprehensive set of fuzzy rules (based on various combinations of the input variables) is presented in matrix form in Table 1. In total, 27 fuzzy rules have been defined to guide the decision-making process.

**Table 1:** Fuzzy rule base.

Rule	Input			Output
	Soil moisture	Temperature	Solar-radiation index derived from light intensity (LDR)	Irrigation duration
1	Dry	Cold	Low	Medium
2	Dry	Warm	Low	High
3	Dry	Hot	Low	Very high
4	Dry	Cold	Medium	Medium
5	Dry	Warm	Medium	High
6	Dry	Hot	Medium	Very high
7	Dry	Cold	High	Low
8	Dry	Warm	High	Medium
9	Dry	Hot	High	Medium
10	Moderate	Cold	Low	Low
11	Moderate	Warm	Low	Medium
12	Moderate	Hot	Low	High
13	Moderate	Cold	Medium	Low
14	Moderate	Warm	Medium	Medium
15	Moderate	Hot	Medium	High
16	Moderate	Cold	High	Very low
17	Moderate	Warm	High	Low
18	Moderate	Hot	High	Medium
19	Wet	Cold	Low	Very low
20	Wet	Warm	Low	Low
21	Wet	Hot	Low	Medium
22	Wet	Cold	Medium	Very low
23	Wet	Warm	Medium	Low
24	Wet	Hot	Medium	Medium
25	Wet	Cold	High	Very low
26	Wet	Warm	High	Very low
27	Wet	Hot	High	Low

These rules are formulated by evaluating the influence of each input variable on the water requirements of the vineyard. The relationship between the output variable (irrigation duration) and the input variables (soil moisture, solar radiation, and ambient temperature) is described as follows:

- **Soil moisture**: As soil moisture increases, the irrigation demand decreases, indicating an inverse relationship between soil moisture and irrigation duration.
- **Solar radiation**: Higher solar intensity leads to increased evaporation from the soil surface, thereby extending the irrigation period needed to maintain optimal moisture levels. Thus, a direct correlation exists between solar radiation and irrigation duration.
- **Air temperature**: Temperature has a similar effect to solar radiation on soil moisture. As temperature rises, so does the need for irrigation, establishing a direct relationship.

In conclusion, within the proposed controller, irrigation duration is modeled as directly related to solar radiation and air temperature and inversely related to soil moisture.

### Fuzzy system processing stages

The fuzzy inference process consists of the following stages:

- **Input fuzzification**: Sensor data are converted into fuzzy values.
- **Fuzzy inference engine**: Based on the defined fuzzy rules, the appropriate output for the water pump status is determined.
- **Output defuzzification**: The final numerical value indicating whether the pump should be turned on or off is computed and executed.

### Membership functions

**Fig. 4:**
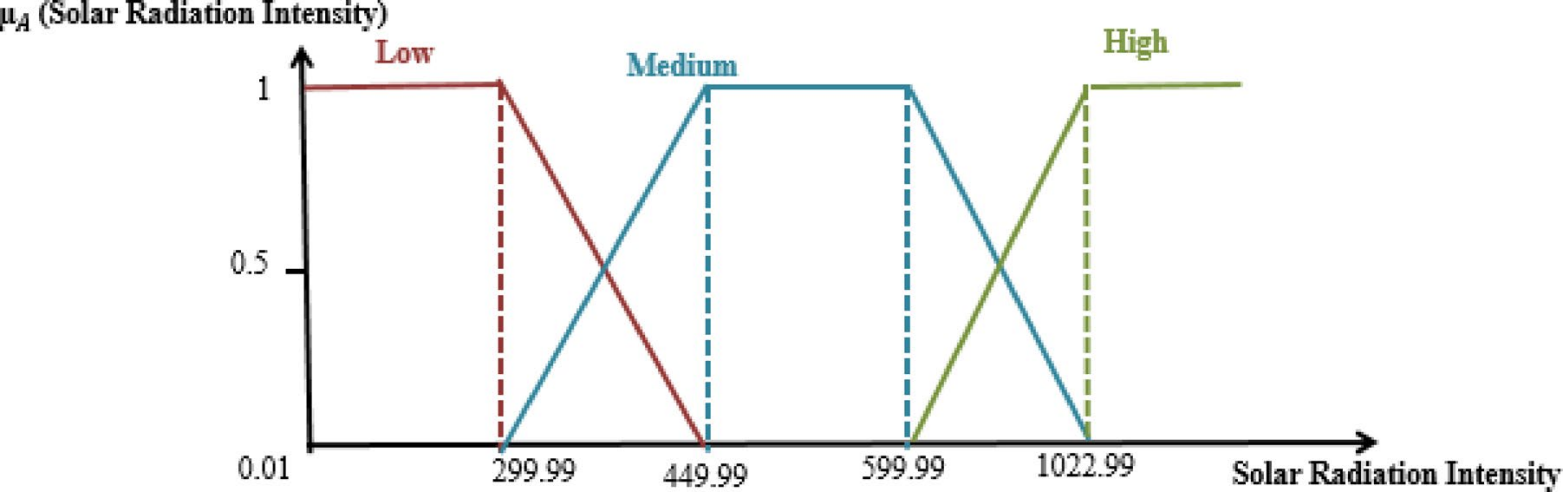
Membership function for the input variable “solar-radiation index derived from light intensity (LDR)”.

**Fig. 5:**
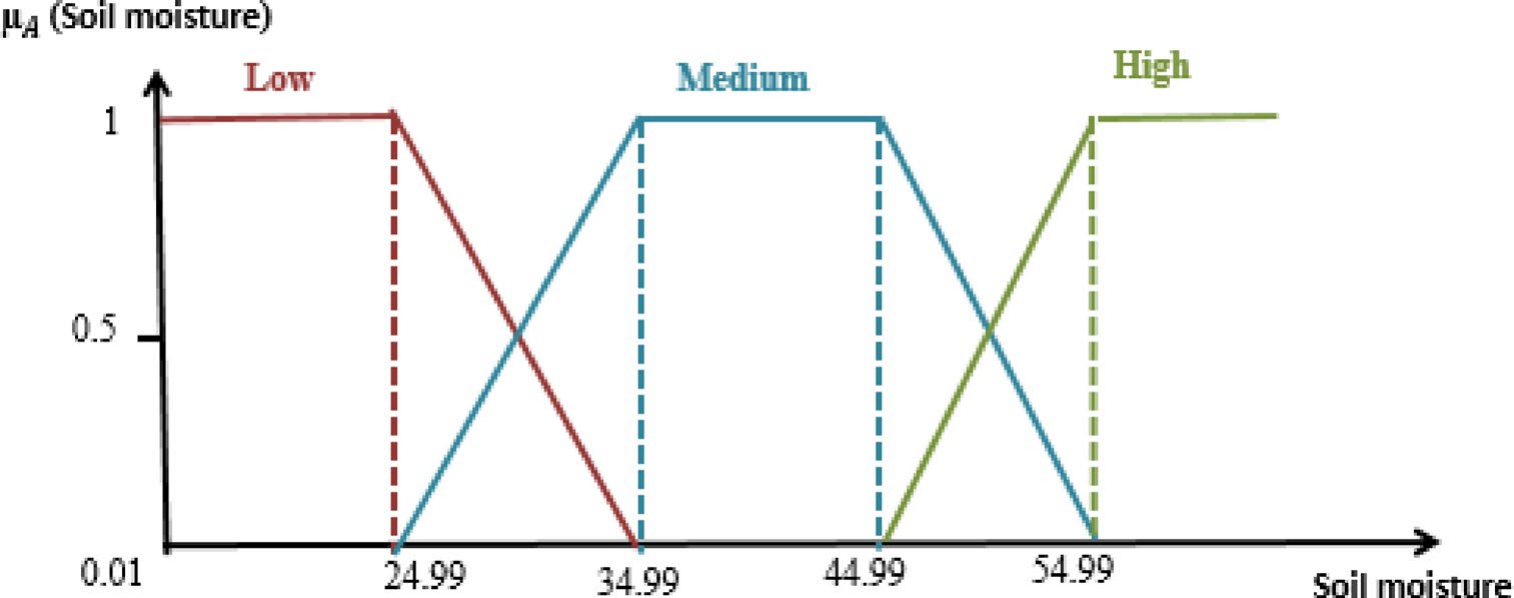
Membership function for the input variable “soil moisture”(% volumetric).

**Fig. 6:**
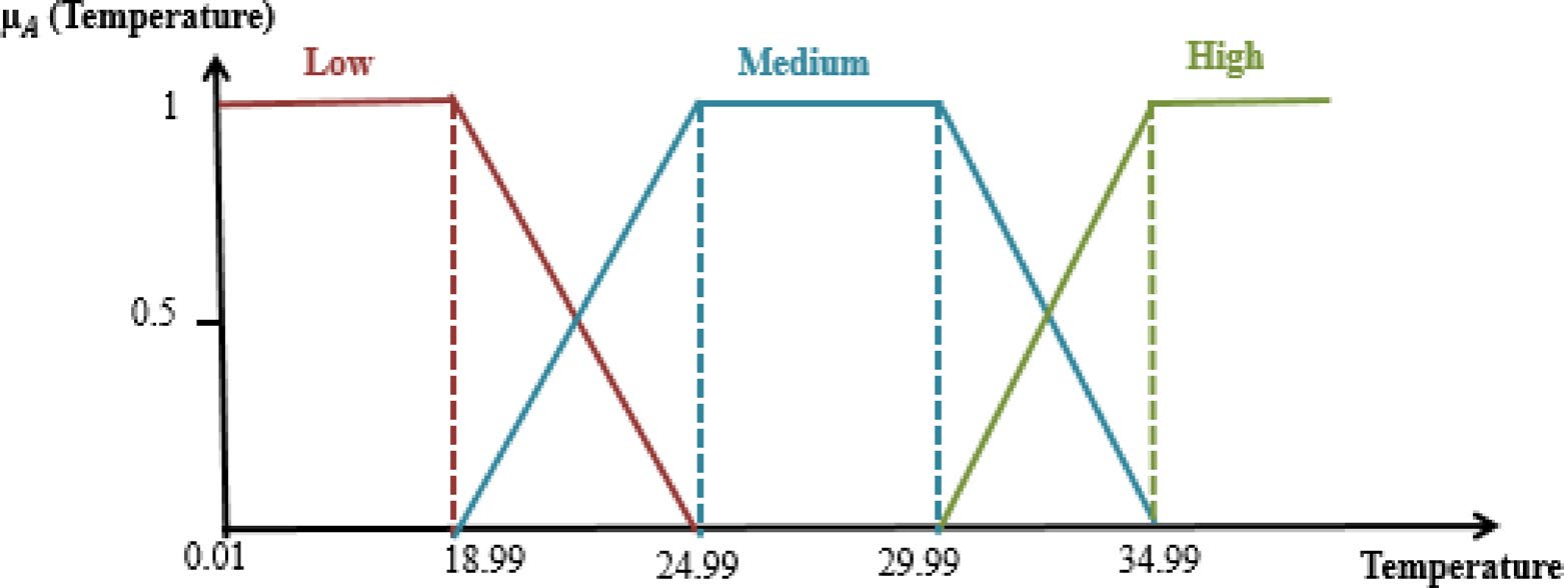
Membership function for the input variable “ air temperature” (°C).

The membership functions for the input variables are illustrated in Figs. 4, 5, and 6. Each input variable is fuzzified using three membership functions corresponding to the fuzzy sets applied in the system.

The membership function thresholds shown in Figs. 4, 5, and 6 were established based on the well-documented physiological requirements of the ‘Bidaneh Keshmeshi’ (syn. Keshmeshi Sefid) grapevine under semi-arid conditions.

**Soil moisture:** Volumetric soil water content below 25% consistently induces moderate-to-severe water stress in grapevines, leading to significant reductions in stomatal conductance and photosynthesis^15^. Accordingly, the fuzzy sets were defined as Low (< 25% = Dry), Medium (25–45%), and High (> 45%), aligned with the available water capacity of the local sandy-loam soil and matching widely accepted critical thresholds for Vitis vinifera.

**Solar-radiation index derived from light intensity (LDR)**: Thresholds of < 300 (Low), 300–600 (Medium), and > 600 (High) were directly derived from in-situ calibration against a reference pyranometer and correspond to low, moderate, and high evaporative demand periods, respectively. Values > 600 represent peak solar radiation (> 800 W m⁻^2^), during which irrigation is minimized or shifted to evening hours to reduce evaporation losses^16^.

**Temperature**: A strong positive correlation exists between air temperature and crop evapotranspiration in vineyards. Temperatures exceeding 30 °C markedly increase vapour pressure deficit and transpirational demand^17^. Consequently, the fuzzy sets were defined as Low (< 18 °C), Medium (18–30 °C), and High (> 30 °C = Hot).

These physiologically grounded thresholds enable the fuzzy inference system to emulate expert agronomic decision-making while simultaneously accounting for the combined effects of soil moisture, temperature, and solar radiation on vine water status, thereby ensuring contextually appropriate irrigation scheduling throughout the growing season.

Following the fuzzification stage, the fuzzy inference engine—responsible for the inference process—applies the predefined fuzzy rules to the three fuzzified input parameters and generates the corresponding fuzzy output (i.e., the irrigation duration).

**Fig. 7:**
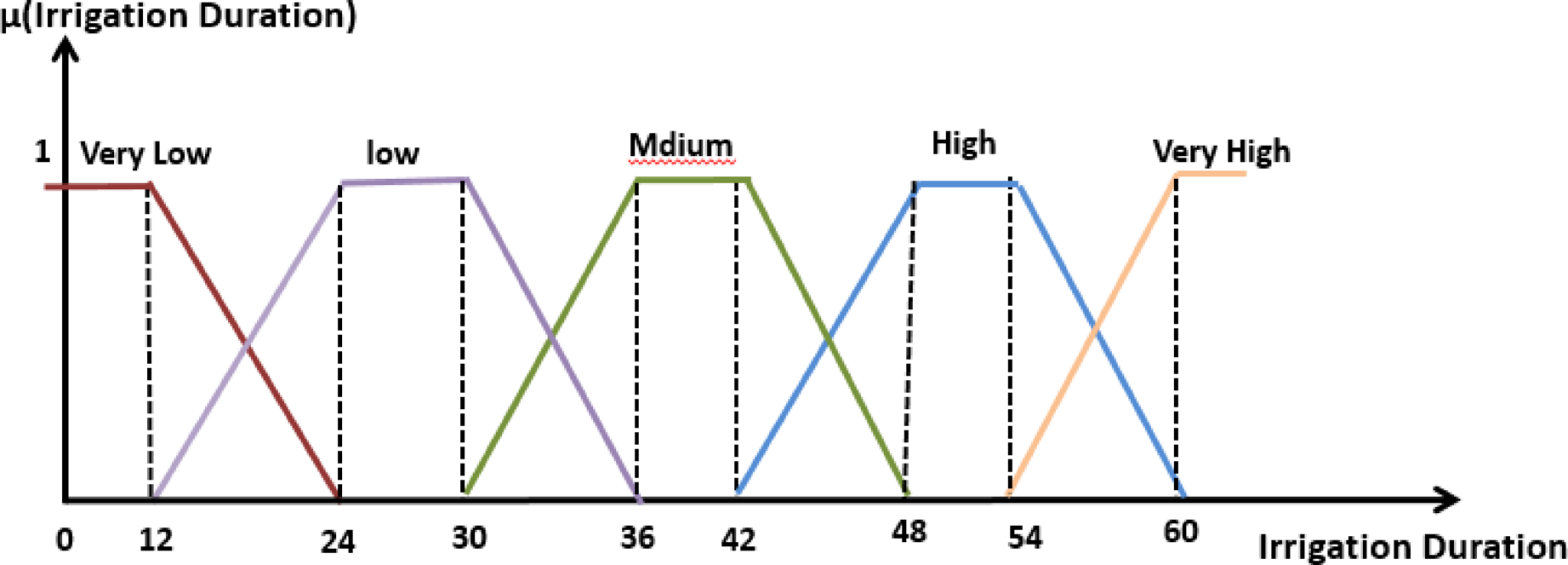
Membership function for the output variable “Irrigation Duration” (minutes).

In practice, once a fuzzy rule is activated, the output associated with that rule is calculated. The defuzzifier then aggregates all fuzzy outputs and converts them into a single crisp numerical value, representing the final irrigation duration. This output is shown in Fig. 7.

### Irrigation water computation and evaluation metrics

The irrigation water volume was computed from pump flow rate and irrigation duration (Equation 2):

3 $$V(m^{3} ) = Q(m^{3} /h) \times \frac{t(min)}{{60}}$$

where $Q = 4.8 m^{3} /h$ and t is the irrigation duration. Seasonal irrigation water use was reported as $m^{3} /ha$ Water saving (%) was computed as Eq. 3:

4 $$Saving(\% ) = 100 \times \frac{{W_{trad} - W_{smart} }}{{W_{trad} }}$$

where $W_{trad}$ and $W_{smart}$ are seasonal irrigation volumes under conventional and smart irrigation, respectively.

### Statistical analysis

Due to the pilot-scale adjacent-plot design and the fact that replicate-level raw measurements (per vine or per cluster) were not retained in a machine-readable format, no inferential statistical tests (e.g., independent t-test or ANOVA) were performed. Results for crop performance indicators are therefore presented as plot-level mean values. To address this limitation in future work, the authors plan to conduct multi-season, randomized block designs with adequate replication, enabling formal hypothesis testing (α = 0.05) and reporting of means + 2.2. Nevertheless, the observed differences (34.6% water saving, 24% increase in cluster weight, 2.2°Brix increase, and 57% reduction in rotten clusters) represent practically significant improvements that warrant further replicated validation.

## Simulation of the proposed method

**Fig. 8:**
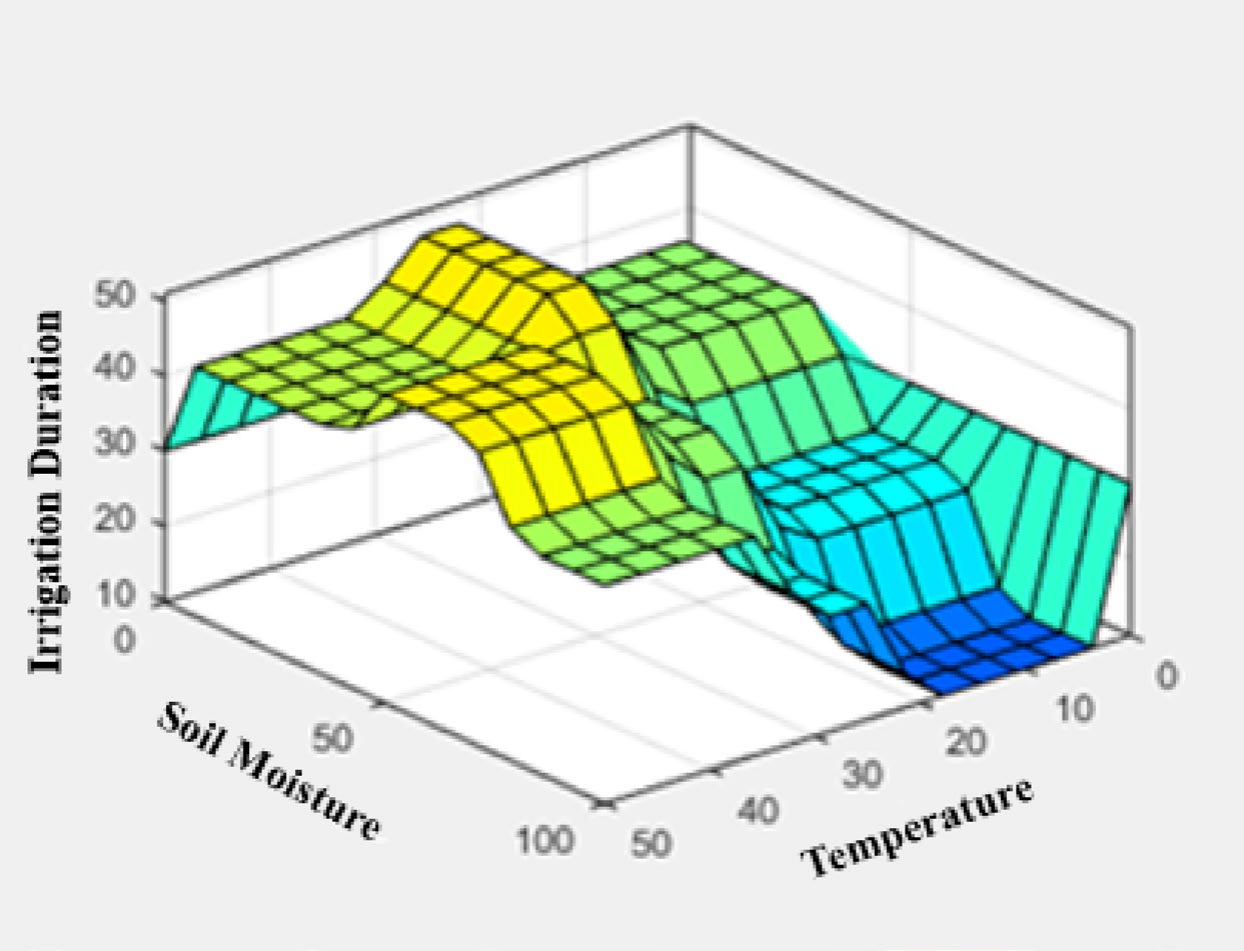
Surface plot illustrating the relationship between soil moisture and air temperature with irrigation duration.

The irrigation system is based on an Arduino microcontroller platform, which receives physical data through sensors connected to the Arduino board. The microcontroller then uses the embedded fuzzy logic code to make decisions regarding irrigation scheduling. Based on predefined fuzzy rules, the system determines the irrigation duration for a one-hectare field using a water pump with a flow rate of 4.8 cubic meters per hour. The proposed method was simulated using MATLAB software. As illustrated in Fig. 8, the surface plot suggests that as soil moisture increases and air temperature decreases, the irrigation duration correspondingly decreases.

**Fig. 9:**
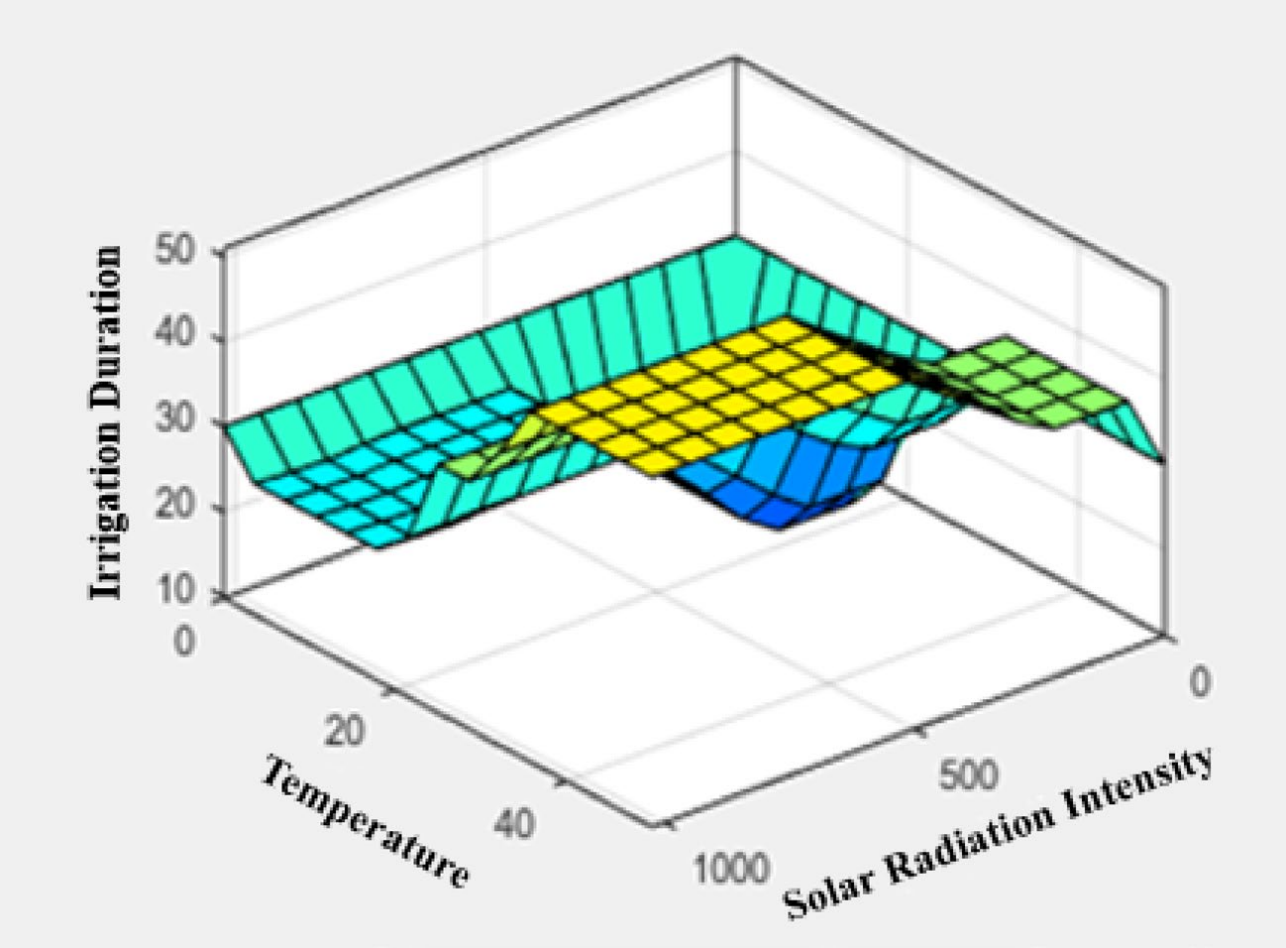
Surface plot illustrating the relationship between temperature and solar radiation with irrigation duration.

**Fig. 10:**
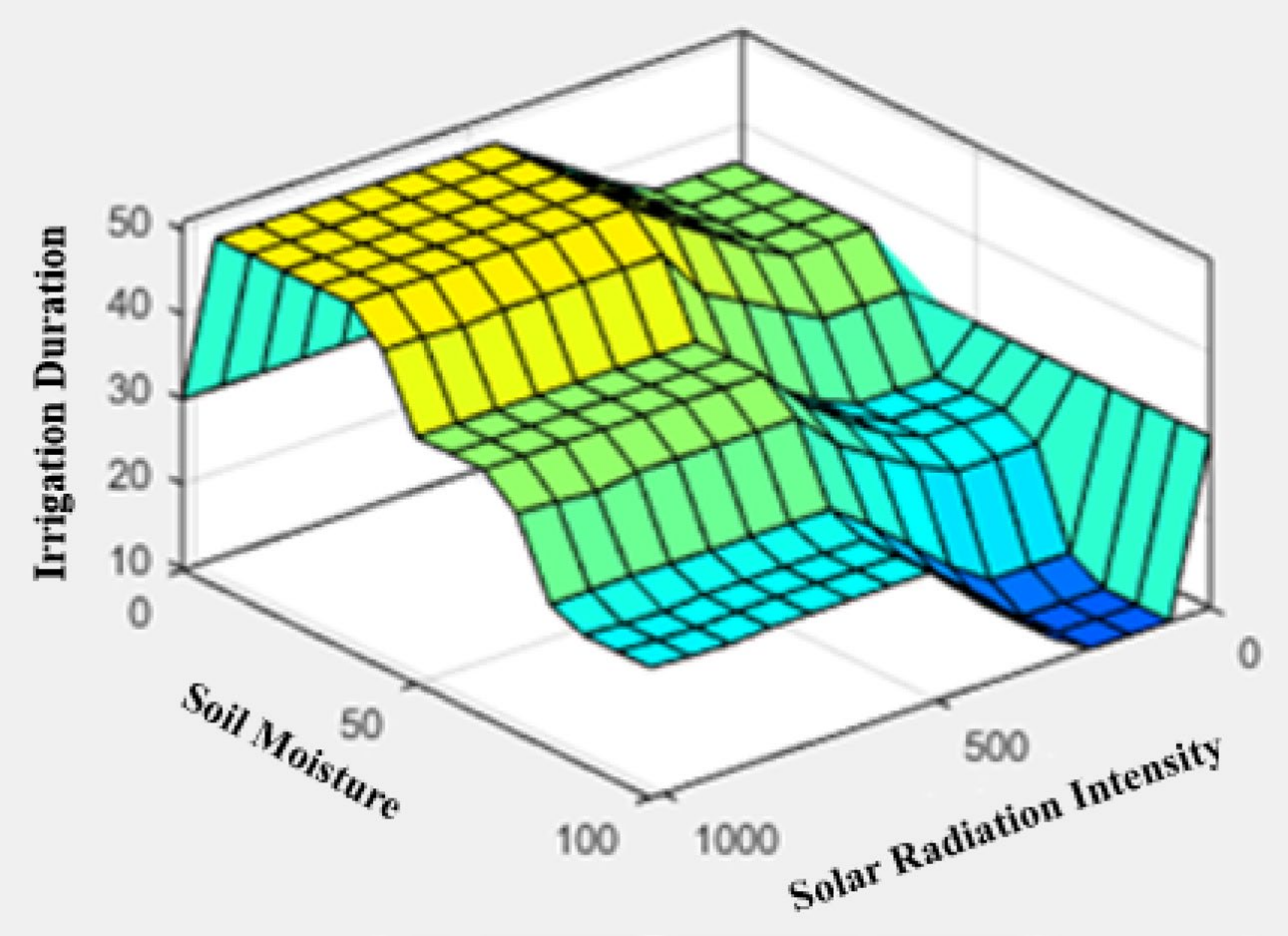
Surface plot illustrating the relationship between soil moisture and solar radiation with irrigation duration.

Moreover, as shown in Figs. 9 and 10, irrigation duration generally increases with higher temperature and lower soil moisture. In practice, the solar-radiation index is also used as an operational cue to avoid irrigation during peak radiation periods in order to reduce evaporation losses; thus, irrigation can be shifted to periods with lower evaporation risk while still meeting crop water needs. These results support the internal consistency of the fuzzy rules used in the controller.

### Field measurements and system performance evaluation

System performance was evaluated using directly measured field variables: soil moisture (%), air temperature (°C), solar-radiation index derived from light intensity (LDR) (0–1023), daily rainfall (mm), irrigation duration (minutes), and pump status (ON/OFF). Sensor readings were acquired every 15 min throughout the growing season. Due to limited onboard memory, representative instantaneous values were manually recorded at approximately 12:00 local time on selected dates (Table 2). Controller performance was assessed by its ability to adjust irrigation duration logically in response to real-time environmental inputs while respecting the rainfall-gating rule. The measured data (Table 2) demonstrated consistent and expected behavior. For instance, under low soil moisture (< 25%), high air temperature (> 34 °C), and high solar-radiation index (> 600), irrigation duration increased progressively to 32–36 min. Conversely, irrigation was correctly suppressed following rainfall detection. These results confirm that the fuzzy rule base effectively translated environmental stress cues into appropriate irrigation decisions. A subsequent agronomic comparison conducted at harvest (September 2024) quantified the benefits of the proposed system in terms of seasonal water savings and grape quality improvement relative to the adjacent conventionally irrigated plot (Table 3).

**Table 2:** Real environmental conditions and smart irrigation system response—March to August 2024, Malekan County.

Date	Air temperature (°C)	Soil moisture (%)	Solar-radiation index derived from light intensity (LDR) (0–1023)	Rainfall (mm)	Pump Status	Irrigation duration (minutes)
Mar 1	12	45	150	0.5	OFF	0
Mar 15	16	40	200	0.0	ON	15
Apr 1	18	38	250	0.0	ON	18
Apr 15	22	35	300	0.0	ON	20
May 1	25	32	350	0.0	ON	22
May 15	28	30	400	0.0	ON	24
Jun 1	30	28	450	0.0	ON	26
Jun 15	33	26	500	0.0	ON	28
Jul 1	35	24	550	0.0	ON	30
Jul 15	38	22	600	0.0	ON	32
Aug 1	36	20	650	0.0	ON	34
Aug 15	34	18	700	0.0	ON	36

**Table 3:** Comparison of grape yield and quality: smart versus traditional irrigation.

Parameter	Traditional irrigation	Smart irrigation
Average cluster weight (grams)	290	360
Brix (sugar content, °Brix)	15.2	17.4
Acidity (g/L tartaric acid)	6.3	5.8
Rotten clusters (%)	7.5	3.2
Irrigation water used (m^3^/hectare)	520	340

### Real-world validation of the smart irrigation system: March to August 2024

To assess operational performance and real-world applicability, the proposed fuzzy logic–based smart irrigation system was deployed in a vineyard in Malekan County (East Azerbaijan Province, Iran) during March–August 2024. This period covers a representative portion of the grapevine growing season, during which environmental conditions (temperature, soil moisture, solar exposure, and rainfall) can vary substantially in a semi-arid climate. The objective of this field pilot was to evaluate whether the controller responds consistently to changing conditions and produces reasonable irrigation-duration outputs.

Field conditions and system responses were monitored throughout the season. Due to the pilot nature of the deployment and limited data retention, Table 2 reports representative dates spanning March–August 2024 to illustrate system behavior under varying environmental conditions. The monitored environmental parameters included air temperature (°C), soil moisture (%), and a solar-radiation index derived from light intensity (LDR) (0–1023) derived from the light sensor after calibration/mapping (higher index values represent higher solar-radiation conditions), as well as daily rainfall (mm). In parallel, the controller outputs (pump status and irrigation duration in minutes) were recorded for the reported dates. When rainfall was detected, the rainfall-gating rule disabled irrigation to prevent unnecessary watering.

Overall, the reported observations indicate consistent and logical controller behavior. For example, on March 1, when air temperature and the solar-radiation index were relatively low and soil moisture was higher (with 0.5 mm rainfall), the system correctly refrained from irrigation (pump OFF). Conversely, during hotter, drier conditions (e.g., July 15 and August 15), characterized by elevated temperatures (> 34 °C), low soil moisture (< 20%), and high solar-radiation index values (> 600), the controller increased irrigation duration (32–36 min). This pattern suggests that the fuzzy inference rules capture expected relationships between environmental stress cues and irrigation demand in vineyard conditions.

### Evaluation of crop performance

As this was an unreplicated pilot-scale adjacent-plot comparison, the observed differences in grape yield and quality parameters should be regarded as exploratory and descriptive rather than statistically confirmed treatment effects.

This study took place in September 2024 in Malekan County, a major grape-producing region in Iran that also experiences increasing water stress. The primary aim was to determine whether irrigation strategies driven by fuzzy logic principles could lead to quantifiable improvements in crop output and quality.

For this purpose, two adjacent vineyard plots were established: one utilizing the proposed fuzzy logic-based smart irrigation system (experimental plot), and the other managed using conventional irrigation techniques (control plot). Key agronomic performance indicators were monitored throughout the trial, including average cluster weight (g), sugar content (°Brix), acidity (g L⁻^1^ tartaric acid), percentage of rotten clusters (%), and total seasonal irrigation water use (m^3^ ha⁻^1^).

These indicators were selected due to their practical relevance in viticulture, as they significantly affect overall productivity, marketability, and post-harvest handling of table grapes.

As this was a pilot adjacent-plot comparison, outcomes are presented descriptively based on plot-level summaries.

The results summarized in Table 3 indicate that the smart irrigation system outperformed the traditional approach in both yield and quality parameters. Specifically, the average cluster weight increased by roughly 24%, suggesting improved vine health and more efficient nutrient assimilation due to optimized water delivery. The increase in Brix value from 15.2 to 17.4 reflects enhanced sugar accumulation and more advanced fruit ripening, while the decrease in acidity is associated with improved flavor balance and longer shelf life—critical traits for both table. One of the most notable findings was a 57% reduction in the proportion of rotten clusters, highlighting better crop health and reduced susceptibility to fungal infections, often triggered by excessive or poorly timed irrigation. The system’s adaptive responses to real-time inputs—particularly soil moisture and ambient temperature—likely contributed to this outcome. Additionally, the innovative system achieved a 34.6% reduction in total water usage, underlining its effectiveness in minimizing resource consumption while maintaining or enhancing productivity. These outcomes collectively suggest the dual advantage of the proposed solution: enhancing agronomic performance while fostering sustainable water use in viticulture. By integrating fuzzy logic with real-time sensor data, the system offers a dynamic, feedback-driven method of irrigation scheduling tailored to the physiological needs of the crop and prevailing climatic conditions. This adaptability is especially valuable in semi-arid and drought-prone regions, where efficient water management is vital. From an agricultural perspective, the system supports higher yields, reduced input costs, and potentially greater economic returns for farmers. More broadly, its implementation aligns with national and global priorities for climate-resilient, resource-efficient farming. Future research should focus on evaluating system performance across diverse grape cultivars and growing seasons to assess generalizability and long-term benefits. Incorporating additional phenotypic traits-such as berry firmness, phenolic composition, and yield per vine-could provide deeper insight into the system’s overall agronomic efficacy and economic impact.

These pilot results should be interpreted with caution because replicate-level measurements and multi-season replication were not available to quantify variability and statistical significance.

## Conclusion

Efficient irrigation control in vineyards is a critical challenge in modern agriculture, particularly in regions facing water scarcity. This study presented a low-cost fuzzy logic–based smart irrigation controller implemented on an Arduino Uno and designed to support water-efficient irrigation decisions in a semi-arid vineyard. The controller integrates real-time environmental inputs—soil moisture, air temperature, and a solar-radiation index derived from an LDR sensor— within a rule-based fuzzy inference framework to determine irrigation duration, while a rainfall-gating rule prevents unnecessary irrigation during and shortly after rainfall events. MATLAB-based surface analyses and field pilot observations from March to August 2024 indicated consistent controller behavior under varying environmental conditions.

In an unreplicated pilot adjacent-plot comparison, the proposed controller was associated with a 34.6% reduction in seasonal irrigation water use (520 → 340 m^3^ ha⁻^1^) and descriptive improvements in plot-level grape performance indicators (cluster weight, °Brix, acidity, and rotten cluster rate) compared with conventional irrigation. Because replicate-level raw measurements and multi-season replication were not available in this pilot implementation, these agronomic outcomes should be interpreted as exploratory and require confirmation through larger-scale, replicated, randomized trials. Overall, the proposed controller provides a practical, low-cost pathway for improving irrigation scheduling in vineyards. Future work should include larger-scale, multi-season, fully replicated field trials to statistically validate these promising pilot results and confirm their generalizability across different cultivars, soil types, and climatic conditions.

## Data Availability

The data that support the findings of this study are available from the corresponding author upon reasonable request.

## Code availability

All code for data analysis associated with the current submission is available from the corresponding author upon reasonable request.
